# Efficacy of a topical formulation containing fipronil, moxidectin and praziquantel (Banni^3®^) for the control of gastrointestinal helminths of zoonotic importance in naturally infected cats

**DOI:** 10.29374/2527-2179.bjvm006025

**Published:** 2025-09-15

**Authors:** Diefrey Ribeiro Campos, Anna Carolina Teixeira de Jesus, Ronald da Silva Souza, Breno Cayeiro Cruz, Gessica Ariane de Melo Cruz, Milenni Garcia Michels, Igor Renan Honorato Gatto, Ferdinando Nielsen Almeida, Thais Ribeiro Correia, Fabio Barbour Scott

**Affiliations:** 1 Departamento de Parasitologia Animal, Instituto de Veterinária, Universidade Federal Rural do Rio de Janeiro, Seropédica, RJ, Brazil.; 2 Ourofino Saúde Animal Ltda, São Paulo, SP, Brazil .

**Keywords:** Anthelmintic, Ancylostoma spp., *Toxocara* spp., Dipylidium caninum, Antihelmíntico. *Ancylostoma* spp., *Toxocara* spp., Dipylidium caninum

## Abstract

Gastrointestinal helminthiases in domestic cats are a significant concern for both animal and human health due to the zoonotic potential of parasites such as *Ancylostoma* spp*.*, *Toxocara* spp*.* and *Dipylidium caninum*. The increasingly close coexistence between humans and cats in urban environments increases the need for effective and practical strategies for parasite control. We evaluated the efficacy of an antiparasitic formulation based on fipronil, moxidectin and praziquantel (Banni^3®^) for topical application. A total of 38 cats infected by the helminths of interest were evaluated and the helminths were found to belong to more than one genus. Coproparasitological evaluations were performed on days D-7 and D-2 (pre-treatment) and D+7 and D+14 (post-treatment) using the modified McMaster technique to count eggs per gram of feces (EPG). In the case of *D. caninum*, proglottids and ovigerous capsules were also analyzed. Of the animals tested, 38 were positive for *Ancylostoma* spp., 20 for *Toxocara* spp. and 22 for *D. caninum.* Six animals had infections by helminths of a single genus, 22 by two and 10 by all three genera. After treatment, the results indicated efficacy of 97.4% (D+7) and 99% (D+14) for *Ancylostoma* spp., 96.12% (D+7) and 97.84% (D+14) for *Toxocara* spp., and 100% (D+7 and D+14) for *D. caninum*. The topical formulation Banni^3^ (fipronil, moxidectin, and praziquantel) demonstrated high efficacy in controlling *Ancylostoma* spp*.*, *Toxocara* spp*.*, and *D. caninum* in naturally infected cats. The results confirm its potential as a practical and effective alternative for the control of zoonotic helminths.

## Resumo

As helmintoses gastrointestinais em gatos domésticos representam uma preocupação relevante tanto para a saúde animal quanto para a saúde pública, devido ao potencial zoonótico de parasitos como *Ancylostoma* spp*.*, *Toxocara* spp*.* e *Dipylidium caninum*. A convivência cada vez mais próxima entre humanos e felinos em ambientes urbanos reforça a necessidade de estratégias eficazes e práticas para o controle desses parasitos. Este estudo avaliou a eficácia de uma formulação antiparasitária tópica à base de fipronil, moxidectina e praziquantel (Banni^3®^). Um total de 38 gatos infectados pelos helmintos de interesse foi incluído na avaliação, podendo apresentar infecção por mais de um gênero de helminto. As avaliações coproparasitológicas foram realizadas nos dias D-7 e D-2 (pré-tratamento) e D+7 e D+14 (pós-tratamento), utilizando a técnica de McMaster modificada para contagem de ovos por grama de fezes (OPG); para *D. caninum*, também foram analisados proglotes e cápsulas ovígeras. Dos animais testados, 38 foram positivos para *Ancylostoma* spp*.*, 20 para *Toxocara* spp*.* e 22 para *D. caninum*; 6 apresentaram infecção por um único helminto, 22 por dois, e 10 por todos os três. Após o tratamento, os resultados indicaram eficácia de 97,4% (D+7) e 99% (D+14) para *Ancylostoma* spp*.*, 96,12% (D+7) e 97,84% (D+14) para *Toxocara* spp*.*, e 100% (D+7 e D+14) para *D. caninum*. A formulação tópica Banni^3®^ (fipronil, moxidectina e praziquantel) demonstrou alta eficácia no controle de *Ancylostoma* spp., *Toxocara* spp. e *D. caninum* em gatos naturalmente infectados. Os resultados confirmam seu potencial como uma alternativa prática e eficaz para o controle de helmintos de importância zoonótica.

## Introduction

Gastrointestinal helminths are of considerable clinical relevance in pets, particularly dogs and cats, due to their impact on animal health, variously causing gastrointestinal disturbances, weight loss, anemia, and in severe cases, death, besides their zoonotic potential (Ballatore Holland Lins et al., 2021). Accordingly, parasites such as *Ancylostoma* spp*.*, *Toxocara* spp*.*, and *Dipylidium caninum* are frequently targeted by public health initiatives and parasite control programs (European Scientific Counsel Companion Animal Parasites, 2021).

These endoparasites are transmitted through diverse pathways, including ingestion of embryonated eggs or infective larvae, active cutaneous or mucosal penetration (particularly in the case of *Ancylostoma* spp*.*)*,* and lactogenic transmission during nursing (Rinaldi et al., 2022). In this context, domestic cats serve as important reservoirs, contributing to environmental contamination and increasing the risk of transmission to other animals and humans (Ferreira et al., 2020). In Brazil, *Ancylostoma* spp*.*, *Toxocara* spp., and *D. caninum* are among the most prevalent gastrointestinal parasites in cats, although other species, such as *Toxascaris leonina*, *Physaloptera praeputialis*, and *Taenia taeniaeformis*, can also occur depending on regional factors (Ballatore Holland Lins et al., 2021; Ferreira et al., 2020; López-Osorio et al., 2020; Rinaldi et al., 2022).

Advances in veterinary pharmacology have led to the development of several anthelmintic classes with proven safety and efficacy for the treatment of parasitic infections in small animals (Zajíčková et al., 2020). Praziquantel is a well-established cestodicidal agent effective against *D. caninum* and *T. taeniaeformis*, whereas moxidectin, a macrocyclic lactone, has demonstrated efficacy against a broad spectrum of nematodes, including *T. cati* and *A. braziliense.* Fipronil, primarily an ectoparasiticide, effectively controls infestations by *Ctenocephalides felis*, thereby contributing to the interruption of the *D. caninum* transmission cycle (Campos et al., 2025).

The combination of these active compounds into a single topical formulation is a practical, broad-spectrum therapeutic strategy capable of targeting both endo- and ectoparasites. This approach is particularly valuable for the management of co-infections, common in feline populations, and may also help delay the emergence of drug resistance (Cutolo et al., 2022). Moreover, the spot-on administration is advantageous in terms of owner compliance and accuracy of dosing (Taylor et al., 2022).

In this context, we evaluated the efficacy of a topical formulation containing fipronil (12.5%), moxidectin (0.83%), and praziquantel (8.3%) (Banni^3®^ - Ourofino Saúde Animal Ltda) in naturally infected domestic cats harboring *Ancylostoma* spp., *Toxocara* spp., and/or *Dipylidium caninum*.

## Material and methods

The study was conducted in the field, in the state of Rio de Janeiro, where the cats were included after confirming positivity for one of the evaluated helminth genera (*Ancylostoma* spp., *Toxocara* spp. or *Dipylidium caninum*), according to the results of coproparasitological exams. The experimental protocol was approved by the relevant ethics committee under CEUA no. 7124141220. The owners of the animals included in the field study signed an informed consent form.

The guidelines followed to determine the efficacy of the tested formulation (Banni^3®^ – Ourofino Saúde Animal Ltda) in the field study were those recommended by VICH GL9 Good Clinical Practices - Scientific guideline (EMA,2000), which recommends the set of actions necessary for the development of a scientific animal study using good clinical practices.

### Inclusion of animals

The study included female and male cats over 5 months old and infected with *Ancylostoma* spp., *Toxocara* spp. and/or *D. caninum*. The animals were included after confirmation of positivity for one of the helminths evaluated, according to the results of the coproparasitological exams.

During the study, all animals were under the responsibility of their owners. Visits could occur within a period of +/- 1 day in relation to the experimental day.

### Helminth diagnosis

The research on cat gastrointestinal helminth eggs was carried out with quantitative coproparasitological tests. The feces were collected and transported from the owner's home to the laboratory according to protocols to avoid contamination of the environment.

The quantitative analysis of eggs per gram of feces (EPG) was performed using the modified McMaster technique (Gordon & Whitlock 1939) to diagnose *Toxocara* spp*. and Ancylostoma* spp*.* In the specific case of *D. caninum*, proglottids found in feces and eggs or ovigerous capsules observed in the McMaster test were analyzed. In the pre-treatment period for inclusion of cats in the study, coproparasitological exams were performed between 2 and 7 days beforehand and the average of parasites in the samples was calculated before the start of treatment.

### Treatment

The animals served as the controls themselves. Negative and positive control groups were not used. Instead, we used only the confirmatory and non-confirmatory results of the parasites in question. The cats received the topical product Banni^3®^ (Ourofino Saúde Animal Ltda), based on fipronil 12.5%, moxidectin 0.83% and praziquantel 8.3%. The formulation was applied according to the volume indicated for each body weight range, on the back of the animals, specifically in the center of the base of the skull (nape region). Animals weighing up to 2.5 kg received the application of 0.3 mL and animals with weight from 2.6 to 7.5 kg received 0.9 mL of the investigated veterinary product.

### Statistical analysis to determine efficacy

The efficacy assessment was calculated based on the following formula: [% Efficacy: (MBT) - MAT) / (MBT) x 100], where MBT represents the arithmetic mean of parasite eggs recovered from feces before treatment and MAT denotes arithmetic mean of parasite eggs recovered from feces after treatment for each assessment time.

For the statistical analysis of the egg reduction rates in the feces of animals treated with the target formulation, the average values of the EPG exams performed 2 and 7 days before treatment was first calculated. After this, the data referring to the pre-treatment means and the data from the tests 7 and 14 days after treatment were transformed into Log10(Count+1) to make the distribution parametric. Then the sample data from two related samples were used to compare the average pre-treatment results with each post-treatment assessment. The significance level considered in all tests was 95% (*p* ≤ 0.05) and the program used was BioEstat 5.3.

### Results

All told, 38 cats were examined and included in the study, 21 females and 17 males, with ages ranging from 5 months to 8 years and weight between 2.0 and 5.4 kg. The characteristics of the participating animals are presented in Table 1 below.

**Table 1:** General data on the cats that participated in the field study to evaluate the efficacy of the investigated product (Banni^3®^–12.5% fipronil +0.83% moxidectin +8.3% praziquantel–Ourofino Saúde Animal Ltda).

**Types of infection**	**Group**	**Number of cats**	**Breed**		**Age (months)**	**Weight (Kg)**
***Ancylostoma* spp*.* + *Toxocara* spp. + *D.caninum***	Positive animals	10	MB (*n*= 10) Siamese (*n*= 0)	Mean (SD)	11.3(13.2)	2,9(0,7)
				Median	6	2,8
				Min-Max	05 - 48	2,0 - 4,5
***Ancylostoma* spp. + *Toxocara* spp.**	Positive animals	10	MB (*n*= 9) Siamese (*n*=1 )	Mean (SD)	6.8(2.1)	2,5(0,5)
				Median	6	2,4
				Min-Max	05 - 12	02 - 3,2
***Ancylostoma* spp. + *D.caninum***	Positive animals	12	MB (*n*= 12) Siamese (*n*= 0)	Mean (SD)	53.7(27.9)	3,7(0,5)
				Median	60	3,5
				Min-Max	06 - 96	2,9 - 4,9
***Ancylostoma* spp.**	Positive animals	6	MB (*n*= 6) Siamese (*n*= 0)	Mean (SD)	30(12.6)	3,5 (1,0)
				Median	30	3,3
				Min-Max	12 - 48	2,5 - 5,4
*Treated		38	MB (*n*= 37 ) Siamese (*n*= 1 )	Mean (SD)	27(26,6)	3.65(0.6)
				Median	12	3.1
				Min-Max	05 - 96	2.0 - 5.4

MB, mixed breed; F, female; M, male; Min, minimum; Max, maximum; sd, standard deviation.

* The treated group contains animals infected with more than one parasite genus.

The animals were grouped according to the type of infection. Of the animals tested, 38 were positive for *Ancylostoma* spp., 20 for *Toxocara* spp. and 22 for *D. caninum.* In turn, six had infections by a single helminth, 22 by two and 10 by all three. Treatment was carried out in a standardized manner for all animals, using the experimental product Banni^3®^ (12.5% fipronil + 0.83% moxidectin + 8.3% praziquantel – Ourofino Saúde Animal Ltda). The analysis of efficacy during the experimental days was conducted individually for each helminth group. The details are in Table 1.

Of the 38 cats positive for *Ancylostoma* spp., there was a significant difference between the pre and post-treatment periods. The efficacy in reducing the EPG values of this helminth varied between 97.4% and 99% at 7 and 14 days post-treatment. For the 20 cats positive for *Toxocara* spp., the efficacies in reducing the number of EPG of this helminth were 96.1% and 97.8% in the evaluations at 7 and 14 days after treatment; and in the 22 cats positive for *D. caninum* there was 100% reduction of the proglottid count between days 7 and 14 after treatment. Details of these results are presented in Table 2.

**Table 2:** Average efficacies according to the tests for *Ancylostoma* spp., *Toxocara* spp. and *D.caninum* during the experimental treatment days, evaluating the efficacy of the experimental product (Banni^3®^ – 12.5% fipronil + 0.83% moxidectin + 8.3% praziquantel – Ourofino Saúde Animal Ltda).

**Group of treated animals**			**Experimental days**			
			**Average (D - 7**	**and D - 2)**	**D + 7**	**D +14**
***Toxocara* spp.**	Positive animals		20		6	5
	Negative animals		0		14	15
	Mean (SD)		580,0 (±325,7)		22.5 (±41.3)	12.5 (±22.21)
	Efficacy (%)		**_ _**		**96.1**	**97,8**
***Ancylostoma* spp.**	Positive animals		38		7	3
	Negative animals		0		31	35
	Mean (SD)		411,1 (±263,4)		10.5 (±23.7)	3.9 (± 3.6)
	Efficacy (%)		**_ _**		**97.4**	**99**
***Dipylidium caninum***	Positive animals		22		0	0
	Negative animals		0		22	22
	Mean (SD)		2,41 (±0,67)		0.00 (±0.00)	0.00 (±0.00)
	Efficacy (%)		_ _		**100**	**100**

sd, standard deviation; min, minimum; max, maximum.

## Discussion

The topical combination of fipronil, moxidectin and praziquantel (Banni^3®^) demonstrated high efficacy in the control of *Ancylostoma* spp., *Toxocara* spp., and *D. caninum* in naturally infected cats. Efficacy exceeded 96% for all evaluated nematodes and reached 100% in the case of *D. caninum*, with consistent reductions observed on Days 7 and 14 post-treatment. These results are noteworthy given the inherent challenges in managing gastrointestinal parasites in feline populations naturally exposed to multiple co-infections.

The high efficacy against *Ancylostoma* spp. and *Toxocara* spp. is aligned with the expected activity of moxidectin, a macrocyclic lactone with broad-spectrum action against gastrointestinal nematodes (Zajíčková et al., 2020). Complete elimination of *D. caninum* is attributable to the cestocidal activity of praziquantel, widely documented in felines (Cutolo et al., 2022). Furthermore, this combination has previously demonstrated efficacy against the flea *C. felis felis* (Campos et al., 2025), the intermediate host of *D. caninum*. Therefore, administration of this formulation may also serve as a preventive measure against *D. caninum* infection by interrupting the transmission cycle and reducing the risk of reinfection. This preventive potential is especially relevant in environments with high flea infestation, where continuous exposure to the intermediate host poses a significant reinfection risk. The consistent therapeutic response between Days 7 and 14 supports the sustained efficacy of the formulation.

Comparatively, previous studies evaluating similar formulations combining praziquantel with eprinomectin, moxidectin and/or (S)-methoprene have reported efficacy rates ranging from 92% to 100% against the same parasites (Cutolo et al., 2022). The results of this study indicate that Banni^3®^ is among the most effective products currently available, with the additional benefit of a single-dose topical administration, which favors treatment adherence by pet owners (Taylor et al., 2022).

Another relevant point is the consistent efficacy observed in animals co-infected with two or three parasite genera. This highlights the efficacy of the product in real-world clinical scenarios, where multiple parasitic infections are common and require broad-spectrum therapeutic approaches (Arisov et al., 2019). The fact that treatment efficacy was not compromised in these cases reinforces the robustness of the formulation.

Despite the promising results, this study has some limitations. The absence of a negative control group precludes a formal comparative analysis, although the before-and-after design, in which each animal served as its own control, is widely accepted in field studies. In addition, the sample size was relatively small, limiting broader inferences about the general feline population. Future studies with larger samples and comparisons with other commercial formulations could strengthen the conclusions presented here.

According to the guidelines of the World Association for the Advancement of Veterinary Parasitology (WAAVP), products demonstrating ≥90% efficacy are classified as effective for parasite control in pet animals (Beugnet et al., 2022). All results observed in this study exceeded that threshold, reaffirming the effectiveness of the tested combination.

Altogether, the findings demonstrate that administration of Banni^3®^ is an effective and practical strategy for the integrated control of gastrointestinal parasites of zoonotic importance in cats. Its broad-spectrum action against nematodes, cestodes and ectoparasites also contributes to the prevention of environmental dissemination of these agents, thereby supporting both animal and public health within a One Health framework.

## Conclusions

The topical formulation containing fipronil, moxidectin and praziquantel (Banni^3®^) demonstrated high efficacy in the control of *Ancylostoma* spp., *Toxocara* spp., and *D. caninum* in naturally infected cats, with results that exceed the efficacy thresholds established by international guidelines. Its combined action against *Ancylostoma* spp., *Toxocara* spp. *Dipylidium caninum* and *Ctenocephalides felis felis* (Campos et al., 2025), along with the convenience of single-dose topical administration, positions this formulation as a promising alternative for the integrated management of gastrointestinal parasites of zoonotic importance. In this context, the use of Banni^3®^ stands out as a promising alternative for direct applicability in veterinary clinical practice.
